# Is the routine pressure dressing after thyroidectomy necessary? A prospective randomized controlled study

**DOI:** 10.1186/1472-6815-8-1

**Published:** 2008-03-20

**Authors:** Patorn Piromchai, Patravoot Vatanasapt, Wisoot Reechaipichitkul, Warinthorn Phuttharak, Sanguansak Thanaviratananich

**Affiliations:** 1 Department of Otorhinolaryngology, Faculty of Medicine, Khon Kaen University, Thailand; 2 Department of Radiology, Faculty of Medicine, Khon Kaen University, Thailand

## Abstract

**Background:**

An acute complication of thyroidectomy is fatal hematoma, which can produce an upper airway obstruction needing immediate intubation or tracheostomy. After neck surgery, we usually apply a pressure dressing with a non-woven, adhesive fabric to reduce bleeding and fluid collection at the operative bed. We conducted a prospective, randomized, controlled study to evaluate a pressure *vs*. a non-pressure dressing after thyroid surgery by monitoring blood and serum in the operative bed.

**Methods:**

We studied 108 patients who underwent 116 thyroid surgeries at Srinagarind Hospital, Khon Kaen University, between December 2006 and September 2007. The patients were randomized to either the pressure dressing or non-pressure dressing group. Ultrasound of the neck was performed 24 ± 3 hours after surgery. The volume of fluid collection in the operative bed was calculated. All patients were observed for any post-operative respiratory distress, wound complications, tingling sensation or tetany.

**Results:**

The distributions of age, sex, surgical indications and approaches were similar between the two groups. There was no statistically significant difference in the volume of fluid collection in the operative bed (*p *= 0.150) and the collected drained content (*p *= 0.798). The average time a drain was retained was 3 days. One patient in the pressure dressing group suffered cutaneous bruising while one patient in the non-pressure dressing group developed immediate hemorrhage after the skin sutures.

**Conclusion:**

Pressure dressing after thyroidectomy does not have any significant impact on decreasing fluid collection at the operative bed. The use of pressure dressing after thyroidectomy may not therefore be justified.

**Trial Registration:**

NCT00400465, ISRCTN52660978

## Background

About 200 cases of thyroidectomy are performed per year at Srinagarind Hospital, Khon Kaen University. An acute complication of this operation can lead to a fatal hematoma due to an upper airway obstruction (in 1 to 2.5 percent of cases) [1-4], requiring either intubation or tracheostomy.

After surgery, we usually apply a pressure dressing using a non-woven, adhesive, fabric to help reduce bleeding and collection of fluid on the basis that it may help to decrease dead space, compress bleeding vessels, decrease the slipping of ligature, the re-opening of cauterized veins, and the oozing area, all of which might precipitate a hematoma at the operative bed. The obvious external evidence of hematoma is a change in the neck contour or bulging skin flaps [5,6]. These signs may be obscured by a widely closed area of the pressure dressing and may delay detection of the hematoma, leading to airway obstruction.

We conducted a prospective, randomized, controlled study to assess the role of a pressure *vs*. non-pressure dressing after thyroid surgery, by monitoring fluid accumulation in the operative bed by ultrasound and by collecting serum in suction drains.

## Methods

Approval was sought from the Khon Kaen University Ethics Committee for Human Research before initiating the study and informed consent was given by the patients. The study was carried out on 108 patients who underwent 116 thyroid surgeries at Srinagarind Hospital, Khon Kaen University, between December 2006 and September 2007. The discrepancy between the number of patients and the number of surgeries was due to 8 patients having undergone completion thyroidectomies for well-differentiated thyroid carcinomas confirmed by histopathology. These 8 patients were randomized as fresh cases with no consideration given to any previous surgery.

We excluded patients (1) with cervical lymph node metastases requiring neck dissection, (2) clinical or laboratory indicators of coagulation disorders or (3) whose bleeding cannot be stopped by ligation and/or cautery or (4) with anaplastic (undifferentiated) carcinoma, which is typically large and invades adjacent structures.

We stratified patients into two groups according to operation field extension. The first group comprised patients who underwent lobectomy or completion thyroidectomy and the second group patients who underwent subtotal or total thyroidectomy. The patients in each group were then randomized to either the pressure or non-pressure dressing group using a computer-generated randomization list with balanced blocks of unequal size. The treatment assignments were contained in sealed, opaque, sequentially-numbered, individual envelopes. After randomization, a nurse, not involved in the surgery, opened the envelope and independently prepared the dressing material. We used conventional knot-tying ligation for medium-sized vessels and the monopolar electrothermal vessel-sealing system for small-sized vessels to achieve vascular control. The operating surgeon was informed of the assignment group after having completed closure of the wound to avoid bias during operation. In the pressure dressing group, gauze was placed throughout the operative area and pressure applied with a non-woven, adhesive fabric (Hypafix^®^); in the non-pressure dressing group, gauze was placed on the incision site and held in place with permeable plastic tape (Transpore^®^).

The closed suction drain, with negative pressure (Radivac^®^), was threaded through a separate wound. A single drain was used in patients who underwent lobectomy or completion thyroidectomy *vs*. two closed suction drains for patients who underwent subtotal or total thyroidectomy.

Volume ultrasound (GE Logiq^® ^9) of the neck was performed after surgery 24 ± 3 hours by the same head and neck radiologist or under his/her supervision. The dressing material was removed at the ward before the patient was taken for ultrasound so as not to apprise the radiologist of the type of dressing. The volume of fluid collection in the operative bed was calculated by measuring the maximum diameter in three dimensions; on the assumption that collection would be in the form of an ellipse or sphere [7]. The volume of fluid collected (in the suction drain) was measured separately. Drains were removed when the fluid collection fell below 10 mL/day. All patients were observed for any post-operative respiratory distress, wound complication, tingling sensation or tetany. The specimens were subjected to histopathological examination for final confirmation of diagnosis. Patients were discharged after drain removal.

The sample-size was calculated based on preliminary data obtained in one month. The 5 patients who underwent thyroidectomy were sent to ultrasound. The mean of fluid volume in surgical bed was 1.53 mL and the standard deviation was 2.5 mL. A sample size of 116 patients was calculated to be able to detect a difference volume of 1.6 mL with a two-sided test with a type 1 error of 5% with a power of 90% and adjusted 5% for dropouts.

Statistical analyses were conducted using Stata (version 9, StataCorp LP, Texas, USA). The normal distributions of data were tested by Kolmogorov-Smirnov test. The effectiveness between the pressure and non-pressure dressing groups were analyzed using the unpaired *t *test or Mann-Whitney U test for continuous variables *vs. *the Pearson's χ^2^-test for categorical variables as appropriate. For all tests, *p *< 0.05 was considered statistically significant.

## Results

Of the 116 thyroid surgeries conducted, there were 15 (12.9%) males and 101 (87.1%) females. The average age of patients was 45.45 years (range, 15–76). Pressure dressings were used for 57 patients and non-pressure dressings for 59. A protocol violation occurred in 2 patients assigned to the pressure dressing group as the surgeon used only one drain instead of two after a subtotal thyroidectomy.

Of the operations performed 65.5% (in 76 patients) were lobectomy. Age and sex were statistically similar between the two groups. The reasons for surgery included: thyroid nodule in 65 (56%) patients, multinodular goiter in 23 (19.8%), Graves' disease in 9 (7.8%) and thyroid carcinoma in 19 (16.4%). The distributions of the surgical indications and approaches were similar between the two groups (Table 1).

**Table 1:** Demographics, diagnoses and types of thyroid surgery performed

	Pressure dressing (*n *= 57)	Non-pressure dressing (*n *= 59)
Demographics		
Age	46.39	44.10
Sex (female) (%)	50 (87.7)	51 (86.4)
Diagnoses		
Thyroid nodule (%)	35 (61.4)	30 (50.8)
Multinodular goiter (%)	12 (21.1)	11 (18.6)
Graves' disease (%)	4 (7.0)	5 (8.5)
Well-differentiated thyroid carcinoma (%)	6 (10.5)	13 (22.0)
Types of thyroid surgery		
Group 1	47	51
Lobectomy	42	38
Completion thyroidectomy	5	13
Group 2	10	8
Subtotal thyroidectomy	4	1
Total thyroidectomy	6	7

On day one post-surgery, the amount of fluid collection in the operative bed was assessed for both groups using ultrasound. The average volume in the pressure *vs. *non-pressure dressing groups was 0.195 and 0.408 mL, respectively. The *t*-test was used to assess for any significant difference in the average amount of fluid collected between the two groups and there was no statistically significant difference in the volume of fluid collected in the operative bed (*p *= 0.150). The average drained content from the pressure *vs. *the non-pressure dressing groups was 59.88 and 62.63 mL, respectively, which was not a statistically significant difference (*p *= 0.798) (Table 2).

**Table 2:** Volume of fluid in the operative bed and the volume of drained fluid

	Pressure dressing (*n *= 57)	Non-pressure dressing (*n *= 59)	*p*-value	95% Confidence Interval of the difference
Volume of fluid in operative bed (mL)	0.1954	0.4083	0.150^a^	-0.69–0.26^a^
Volume of drains fluid				
Day 1 (mL)	52.07	53.98	0.156^a^	-10.01–6.18^a^
Day 2 (mL)	7.28	7.03	0.877^a^	-3.75–4.24^a^
Day 3 (mL)	0.53	1.36	0.087^a^	-2.73–1.07^a^
Total (mL)	59.88	62.63	0.798^a^	-13.82–8.32^a^

*P *< 0.05 was considered significant

^a ^Calculated with unpaired *t*-test

The average time a drain was retained was 3 days and the average hospital stay was 5 days. There was no statistically significant difference between groups (*p *= 0.225 and 0.543, respectively). One patient in the pressure dressing group suffered cutaneous bruising; while an immediate hemorrhage after skin suture developed in one patient from the non-pressure dressing group. In the latter case, we re-explored and found ligation material was dislodged from anterior jugular vein. No patient had any post-operative respiratory distress, tingling sensation or tetany (Table 3).

**Table 3:** Duration of retained drains, complications and length of hospital stay

	Pressure dressing (*n *= 57)	Non-pressure dressing (*n *= 59)	*p*-value	95% Confidence Interval of the difference
Duration of retained drains (days)	2.32 ± 0.51	2.36 ± 0.71	0.225^a^	-0.27–0.19^a^
Complications				
Cutaneous bruising	1	0	1.044^b^	
Hemorrhage	0	1	0.975^b^	
Hospital stay (days)	4.37 ± 0.52	4.37 ± 0.69	0.543^a^	-0.23–0.22^a^

*P *< 0.05 was considered statistically significant

^a ^Calculated using the unpaired *t*-test

^b ^Calculated with the chi-square test

## Discussion

Hematoma is a rare complication of thyroid surgery but the condition can cause an upper airway obstruction, albeit rare (range, 1 to 2.5 percent). The etiology includes slipping of ligature on major vessels (which occurred in this study), reopening of cauterized veins, retching, vomiting, bucking during recovery, valsalva maneuver, increased blood pressure during recovery, large dead space and oozing from the cut area of the thyroid [6]. In our institute, the surgeon usually applies a pressure dressing on the basis that it may help to decrease dead space, compress bleeding vessel and limit the oozing area. Furthermore, it prevents the patient from moving the neck freely which may help to decrease slippage of the ligature or reopening of cauterized veins.

Although many surgeons have gone beyond abandoning a pressure dressing to completely avoiding the use of a drain [8,9], to our knowledge there has been no prospective randomized study to prove the benefits of pressure dressing in post-thyroidectomy patients. We designed our study to use ultrasonography of the neck to measure fluid collection in the thyroid bed to provide an objective assessment of a method commonly used in many reports [10,11]. The weakness of our study was that we did not have an objective control of pressure on the skin after applying the pressure or non-pressure dressing. This problem was discussed among the investigators before initiating the study, and we decided not to use the pressure meter because we wanted to make the study approximate regular practice.

Our average hospital stay was slightly longer (5 days) than that of other institutions (3–6 days)[12,13], perhaps due to the variable definition of hospitalization and the criteria for discharging patients in each study. We admitted patients 1 day prior to surgery and we counted the day we discharged the patient (usually in the morning) as one day. In uncomplicated cases, the patients were discharged from the hospital after drain removal.

In our study, when using a pressure dressing over against a non-pressure dressing, fluid collection at the operative bed and collected drained content was lower in the pressure dressing group. This matched our hypothesis but there was no significant difference. Considering theory on the late detection of hematoma in pressure dressing due to a widely closed area, we did not find either immediate or late hematoma in this group. We suggest that the pressure dressing may have a benefit for preventing hematoma but a larger, randomized, controlled trial is required to establish this practice.

## Conclusion

Pressure dressing after thyroidectomy does not provide any significant benefit vis-à-vis decreasing fluid collection at the operative bed. The use of a pressure dressing after thyroidectomy may, therefore, not be justified.

## Competing interests

The author(s) declare that they have no competing interests.

## Authors' contributions

PP designed and supervised the research; collected, analyzed, and interpreted the data; and drafted and revised the manuscript. PV contributed to the conceptual design of the study. WR contributed to the conceptual design and coordinated the project. WP did the ultrasonographic assessments. ST made substantial contributions to the design of the study and approved the final version of the manuscript. All authors read and approved the final manuscript.

## Pre-publication history

The pre-publication history for this paper can be accessed here:


